# Damage Evolution and Fracture Events Sequence Analysis of Core-Shell Nanoparticle Modified Bone Cements by Acoustic Emission Technique

**DOI:** 10.3390/polym12010208

**Published:** 2020-01-15

**Authors:** O.F. Pacheco-Salazar, Shuichi Wakayama, L.A. Can-Herrera, M.A.A. Dzul-Cervantes, C.R. Ríos-Soberanis, J.M. Cervantes-Uc

**Affiliations:** 1 Instituto Tecnológico Superior de Calkiní en el Estado de Campeche, Avenida Ah Canul, s/n por Carretera Federal, C.P. 24900, Calkiní, Campeche, Mexico; maadzul@itescam.edu.mx; 2 Department of Mechanical Engineering, Tokyo Metropolitan University, 1-1 Minami-Osawa, Hachioji-shi, Tokyo 192-0397, Japan; wakayama@tmu.ac.jp; 3 Departamento de Física Aplicada, CINVESTAV-IPN, Unidad Mérida, Carretera Antigua a Progreso km 6, Cordemex, C.P. 97310, Mérida, Yucatán, Mexico; luis.can.herrera7@gmail.com; 4 Unidad de Materiales, Centro de Investigación Científica de Yucatán, A.C., Calle 43 No. 130 x 32 y 34, Chuburná de Hidalgo, C.P. 97205, Mérida, Yucatán, Mexico; rolando@cicy.mx (C.R.R.-S.); manceruc@cicy.mx (J.M.C.-U.)

**Keywords:** bending test, bone cement, acoustic emission, core-shell nanoparticles

## Abstract

In this research, damage in bone cements that were prepared with core-shell nanoparticles was monitored during four-point bending tests through an analysis of acoustic emission (AE) signals. The core-shell structure consisted of poly(butyl acrylate) (PBA) as rubbery core and methyl methacrylate/styrene copolymer (P(MMA-*co*-St)) as a glassy shell. Furthermore, different core-shell ratios 20/80, 30/70, 40/60, and 50/50 were prepared and incorporated into the solid phase of the bone cement formulation at 5, 10, and 15 wt %, respectively. The incorporation of a rubbery phase into the bone cement formulation decreased the bending strength and bending modulus. The AE technique revealed that the nanoparticles play an important role on the fracture mechanism of the bone cement, since a higher amount of AE signals (higher amplitude and energy) were obtained from bone cements that were prepared with the nanoparticles in comparison with those without nanoparticles (the reference bone cement). The SEM examination of the fracture surfaces revealed that all of the bone cement formulations exhibited stress whitening, which arises from the development of crazes before the crack propagation. Finally, the use of the AE technique and the fracture surface analysis by SEM enabled insight into the fracture mechanisms that are presented during four-point bending test of the bone cement containing nanoparticles.

## 1. Introduction

Acrylic bone cements, which are polymeric materials that are tolerated by the human body, have been employed in clinical applications to fix joint prosthesis, such as the hip and knee. They are mainly used to achieve mechanical stability of the implant and adapt the surface irregularities of the surrounding bone tissue to the surface of the inserted prosthesis [1]. While the bone cements transfer the body weight from the prosthesis to the bone under the service conditions, they undergo loads approximately three times body weight when walking and eight times body weight when stumbling [2]. Thus, the bone cements are vulnerable to mechanical failure, e.g., the loss of mechanical integrity and aseptic loosening of cemented joint implants, which was mainly caused by the compression, tension, and shearing stresses of the loads present during the fracture process [3,4].

Researches to explore the fracture mechanisms of the bone cements by monitoring in real time under the mechanical stresses are limited. The Acoustic Emission (AE) technique is a nondestructive technique and it would provide insight into the failure mechanisms of the materials. In general, this technique is used to alert the system in initial stages of the damage by continuous monitoring through piezoelectric sensors, which integrate on the specimen and receive the high-frequency elastic waves that are produced by the fracture process [5,6].

Numerous studies have been carried out on commercial bone cements while using the AE technique for fatigue loading mainly [7]. For example, Roques et al. [8] employed the AE technique to monitor the damage in the CMW-1 bone cement during four-point bending fatigue tests, recording a good correlation between the AE source and the crack locations. On the other hand, Ng and Qi [9] monitored the damage in the Palacos R bone cement using compact tension specimens during fatigue loading. They developed a wavelet-based acoustic emission (WBAE) technique that was useful in identifying and eliminating the noises from AE signals. Furthermore, Jeffers et al. [10] and Sinnett-Jones et al. [11] recorded the AE signals presented during uniaxial tensile fatigue tests that were performed on CMW-1 bone cement. Both of the authors recognized that the porosity is one of the factors that cause a large amount of AE signals during the tests and control the fatigue life of cements. All of these studies have shown promising results in identifying the fracture mechanisms of the bone cements during cyclic loading by the AE technique. 

In contrast, there has been limited research performed regarding the fracture mechanisms of bone cements under static (also called quasistatic) stresses while using the AE technique. For example, Rios-Soberanis et al. [12] monitored the damage in bone cements modified with a secondary monomer in their chemical formulation during a quasistatic tensile test by the AE technique, recording an obvious correlation between the AE signals and the monomer concentration. 

On the other hand, increasing in vivo longevity of acrylic bone cement by improving the resistance to failure of the polymer has received some attention. There has been great enthusiasm and optimism in investigating the potential of core-shell nanoparticles to enhance the fracture toughness and reduce the elastic modulus in the bone cement [13,14]. Recently, the effect of the incorporation of core-shell nanoparticles on the fracture mechanisms of the acrylic bone cement during the quasistatic compression [14], as well as during the tensile fatigue tests [15] by the AE technique has been published. However, a study, which detailed on damage accumulation of bone cement that was prepared with core-shell nanoparticles during four-point bending tests through an analysis of acoustic emission signals, has not been reported. The bending forces, which represent a combination of tension and compression stresses, are mainly presented in the artificial hips [2].

Therefore, the aim of the present work was to shed light on the fracture mechanism of the bone cements through monitoring the damage accumulation in the bone cements that were prepared with core-shell nanoparticles during the four-point bending test by analyzing the acoustic emission signals. The core-shell structure consisted of poly(butyl acrylate) (PBA) as a rubbery core and random copolymer of poly(methyl methacrylate-*co*-styrene) (P(MMA-*co*-St)) as glassy shell, while considering that the powder component of Surgical Simplex® P bone cement contains this copolymer. The powder component of this commercial bone cement contains P(MMA-*co*-St) at 73.5 wt %, poly(methyl methacrylate) (PMMA) at 15 wt %, barium sulfate (BaSO_4_) at 10 wt %, and benzoyl peroxide (PBO) at 1.5 wt %, whereas the liquid component consists of methyl methacrylate monomer (MMA) at 97.4 vol % and *N*,*N*-Dimethyl-p-toluidine (DMPT) at 2.6 vol % [16]. Furthermore, core-shell ratios 20/80, 30/70, 40/60, and 50/50 were prepared and then incorporated into the solid phase of the bone cement formulation at 5, 10, and 15 wt %.

## 2. Materials and Methods

### 2.1. Materials

Core-shell nanoparticles in this research were synthesized while employing methyl methacrylate (MMA), butyl acrylate (BA), and styrene (St). All of the monomers were purchased from Aldrich, Milwaukie, Wisconsin, USA (99% pure), and then purified by passing through a column filled with basic alumina to remove the inhibitors. The initiator potassium persulphate (K_2_S_2_O_8_), the surfactant sodium dodecyl sulfate (SDS), and the cross-linking agent ethylene glycol dimethyl acrylate (EGDMA) were also acquired from Aldrich, 99% pure, and used without further purification. Distilled and deionized water were employed during the reaction (Agua destilada del sureste, Mérida, México).

### 2.2. Core-Shell Nanoparticles Synthesis

Nanoparticles at different core-shell ratios (20/80, 30/70, 40/60, and 50/50) were prepared by two-stage seeded emulsion polymerization by the growth of PBA seed latex previously polymerized. The synthesis was performed in a two-liter four-necked round bottom glass reactor that was equipped with a condenser, a mechanical stirrer, and a gas inlet to maintain a nitrogen atmosphere. The reactor was immersed in a water bath with thermostatic control to maintain the temperature at 80 °C. 

Emulsion polymerization synthetized the PBA seed. For this purpose, 350 mL of distilled water and 2 g of SDS were added to the reactor. When the temperature was stable at 80 °C, 175 g of BA and 1.75 g of EGDMA were charged to the system, and the emulsion polymerization was initiated by the addition of 1.75 g of initiator that was dissolved in 30 mL of distilled water. The polymerization was completed after three hours of reaction time.

The PBA core was similarly prepared by emulsion polymerization. For this stage, 350 mL of distilled water, 1.5 g of SDS and PBA seed previously polymerized were filled into the reactor at 80 °C. After an aqueous solution of initiator (1 wt %) was added, the BA monomer was incorporated to the system at an addition rate of 0.5 mL/min. The reaction time was two hours under continuous mechanical stirring. 

The P(MMA-*co*-St) shell were obtained during a second stage of polymerization by adding the PBA core formed in the previous stage and an aqueous solution of initiator (1 wt %) into the reactor and mixed for 10 min at 80 °C. Subsequently, a mixture of MMA and St monomers was added dropwise (0.5 mL/min). The core-shell nanoparticles polymerization was completed after two hours of reaction.

The obtained core-shell latex was dispersed in distilled water to decrease the agglomeration of nanoparticles during the precipitation. Afterwards, the nanoparticles were precipitated from the latex by a defrosted process. Finally, the powdered core-shell nanoparticles were washed to eliminate adsorbed surfactant and unreacted monomer, dried in an oven at 60 °C for 24 h, ground, and then sieved (No. 170 Mesh). The different core-shell nanostructures were obtained by incorporating a different percentage of monomer during each stage.

Characterization by Transmission Electron Microscopy (TEM) and Scanning Electron Microscopy (SEM) showed that most of the core-shell nanoparticles exhibited well-defined spherical morphology with an average diameter of 125 nm. Higher agglomerates were formed when the PBA content was increased. The Dynamic Mechanical Analysis (DMA) revealed that the core and shell phase in the different nanoparticle compositions were correlated with the reduction in storage modulus and the maxima of tan δ, as reported elsewhere [13,14].

### 2.3. Bone Cement Preparation

Table 1 shows the bone cement formulations, which were prepared by hand mixing the powder and liquid components without vacuum, while employing an approximate powder-liquid ratio of 2:1 by weight in all cases. The powder component of reference bone cement consisted of PMMA beads, Nic Tone brand (Manufacturera Dental Continental, Guadalajara, México), barium sulfate (BaSO_4_), and benzoyl peroxide (BPO), whereas the liquid component consisted of methyl methacrylate monomer (MMA) and *N*,*N*-Dimethyl-p-toluidine (DMPT) (Aldrich, Milwaukie, WI, USA). On the other hand, the powder component of bone cement containing core-shell nanoparticles was prepared by replacing 5, 10, and 15 wt % of the PMMA beads in the reference bone cement with different core-shell nanoparticles (20/80, 30/70, 40/60, and 50/50), whereas the liquid component remained unchanged.

**Figure 1:**
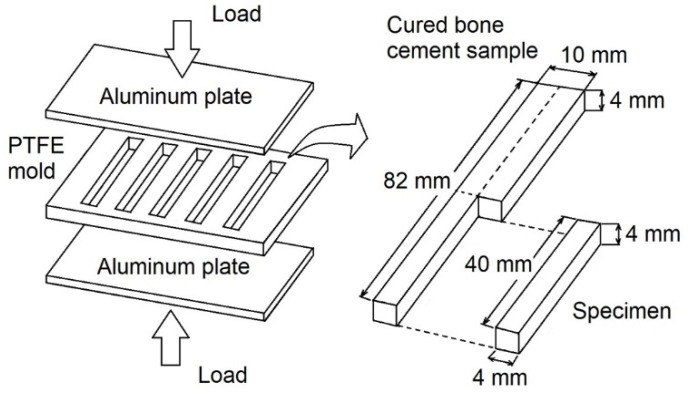
Schematic representation for the preparation of four-point bending test specimens.

Figure 1 presents a schematic illustration for the preparation of four-point bending test specimens. The doughy mixture obtained by mixing the solid and liquid components was placed into a polytetrafluoroethylene (PTFE) mold. Subsequently, the mold was placed in a carver press, model C (Carver, Inc., Wabash, Indiana, USA), at room temperature until the bone cement fully cured, while using a one-ton pressure. Finally, the samples were cut using a cutter machine RC-120 (Ritoku co., LTD, Tokyo, Japan) from cured bars and then polished (sandpaper No. 2000). The employed dimensions were tested instead of those that were recommended by ISO 5833 standard [17] to ensure a better record of AE signals during the test; it has been reported that acrylic bone cements show an AE signal attenuation of 0.2 dB/mm [8], being more sensible for energy than for amplitude [18].

### 2.4. Four-Point Bending Test Setup

**Figure 2:**
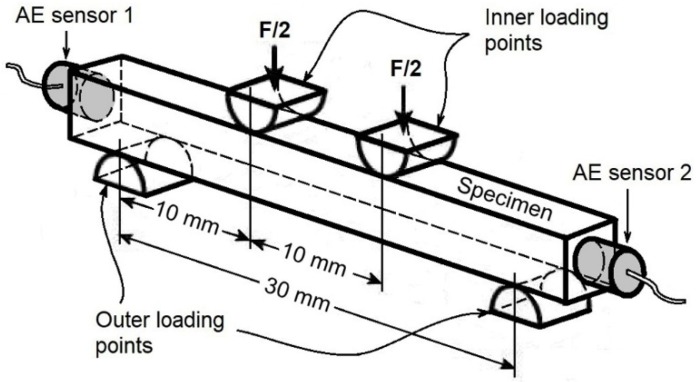
Schematic diagram of the experimental setup showing the four-point bending test and the locations of the Acoustic Emission (AE) sensors on the specimen.

The four-point bending test was carried out in a Shimadzu AG-25TC (Shimadzu, Kyoto, Japan) testing machine in air at room temperature (25 °C). Bending mechanical tests were performed at a crosshead speed of 0.1 mm/min, instead of that recommended by ISO 5833 standard (5 mm/min), in order to achieve a better correlation between the AE signals and the deformation. That is, the specimens were smaller than those that were established by the standard, causing the fracture to occur at a smaller deformation. The distance employed between outer loading points was 30 mm, while the distance utilized between inner loading points was 10 mm (Figure 2).

According to ISO 5833 standard, for each test strip, the bending modulus, E, was calculated from the expression:

E = [35a × (3l^2^ − 4a^2^)]/4fbh^3^

where f is the difference between the deflections under the loads of 15 N and 50 N, in millimeters; b is the average measured width of strip in millimeters; h is the average measured thickness of strip in millimeters; l is the distance between outer loading points (30 mm); and, a is the distance between the inner and outer loading points (10 mm).

The bending strength, B, was calculated from the expression:

B = 3Fa/bh^2^

where F is the force at break in newtons.

The average values of bending modulus and bending strength were calculated for four specimens being expressed in megapascals and the standard deviation.

### 2.5. Acoustic Emission Technique

The AE technique monitored the damage accumulation in bone cement formulations during the four-point bending test. Two AE sensors, type Z3T3D-LYX (Fuji ceramics corporation, Shizouka-ken, Japan), with a resonant frequency of 450 kHz were mounted on the specimen ends by using a cyanoacrylate glue. For each sensor, a preamplifier amplified the detected AE signals (Gain: 60 dB) through the band pass filter with a range of 95 kHz to 660 kHz; the threshold level was 30 dB (31.6 µV). All of the acoustic emission signals and their associated parameters were recorded and analyzed throughout the experiment by using an AE analyzer Vallen Systeme, AMSYS-5 (Vallen Systeme GmbH, Icking, Germany). 

The amplitude and energy were used as damage parameters. Amplitude is the highest peak voltage that was attained by an AE waveform. This is a very important parameter because it directly determines the AE event. Acoustic emission amplitudes are directly related to the magnitude of the source event, and they are customarily expressed on a decibel (logarithmic) scale. However, the measured area under the rectified signal envelope (MARSE), which is sometimes known as energy counts, is the preferred parameter over counts, because it is sensitive to amplitude as well as duration, and it is less dependent on threshold setting and operating frequency. The total AE activity was measured by summing the magnitudes of all the detected events (cumulative energy); among all the measured parameters, MARSE was the one best [19].

On the other hand, the wave velocity in the material was determined by breaking a graphite pencil lead in order to calculate the AE sources location on the specimen during the test. The pencil lead fracture is the most widely used simulated AE source, because of its simplicity, reproducibility, and good time response [20,21]. The breaking of the lead creates a very short-duration, localized impulse that is quite similar to a natural acoustic emission source, such as a crack (known as a Hsu-Nielsen source). Furthermore, the amplitude of lead break source is well within the range of typical crack source [22]. Thus, measuring the distance and recording the time taken for an elastic wave to arrive from the source to the sensors, the wave velocity in the different bone cement formulations was determined, where the arrival time was recorded by using the AE analyzer Vallen Systeme, AMSYS-5. Once this was known, a hit could be located between the two sensors, being based on the time lag between arrivals at each sensor.

Finally, the surface analysis of the specimens tested under four-point bending was carried out while using a Scanning Electron Microscope (SEM), JEOL 6360LV (JEOL USA, Inc., Peabody, MA, USA). The analysis was conducted on the fracture surface that was obtained at the end of the mechanical test; specifically, between the inner loading points (Figure 2). For this purpose, the fractured surfaces were cut and then deposited over metallic supports while using graphite tape. Subsequently, they were coated with a thin layer of gold while using an ion sputtering Delton vacuum, model desk II (Denton Vacuum LLC, Moorestown, New Jersey, USA) before SEM examination.

## 3. Results and Discussion

### 3.1. Four-Point Bending Test

**Figure 3:**
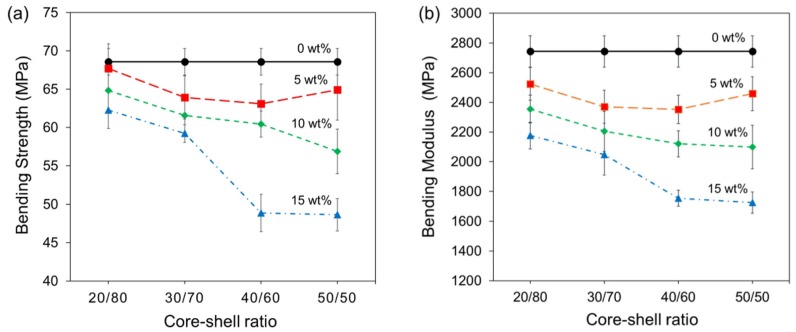
Four-point bending properties of bone cement formulations: (**a**) bending strength and (**b**) bending modulus. Error bars represent the calculated standard deviation.

Figure 3 shows the four-point bending properties of bone cement formulations. It is noted that the bending strength and bending modulus both decreased when the nanoparticles content increased in the bone cement formulation, whereas the core-shell ratios did not seem to cause a really significant change in the bending properties. A two-way analysis of variance (ANOVA) with means comparison (Tukey test) was performed and showed that the reduction of bending properties was only statistically significant when the core-shell ratio 40/60 and 50/50 were employed at 15 wt % concentration. Therefore, the nanoparticles concentration is the principal factor that controls the bending properties of cements in comparison to the different core-shell ratios. The decrease that was observed in the bending modulus has been attributed to the presence of the rubbery cores that posses a glass temperature lower than the room temperature, while the reduction that was presented in the bending strength could be explained by the poor interfacial adhesion between the core-shell nanoparticles agglomerates and the acrylic matrix, such as it was proved by the authors for the quasistatic compression tests [14] as well as for the tensile fatigue tests [15].

All bone cement formulations fulfilled with the minimum bending strength (50 MPa) and the minimum bending modulus (1800 MPa) established for the standard ISO 5833, except for the formulations containing 15 wt % of nanoparticles 40/60 and 50/50 [17].

### 3.2. Acoustic Emission Signals Results

**Figure 4:**
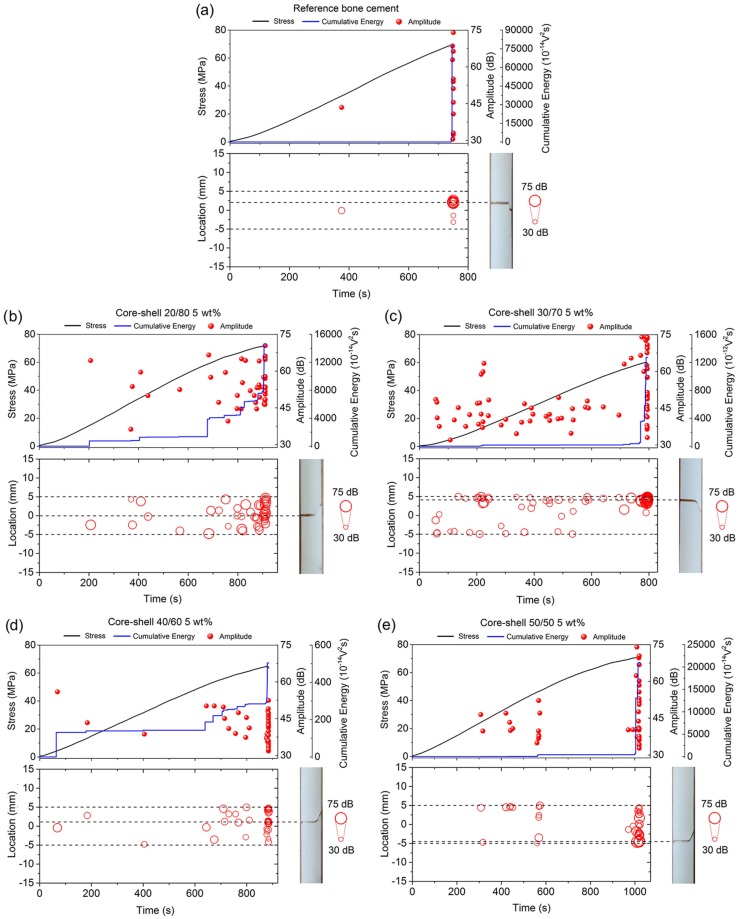
AE signals detected during the four-point bending test in correlation to their respective locations along the specimen surface in (**a**) the reference bone cement and bone cements containing 5 wt % of core-shell nanoparticles (**b**) 20/80, (**c**) 30/70, (**d**) 40/60, and (**e**) 50/50.

Figure 4 shows the AE signals that were detected during the four-point bending test in correlation to their respective locations along the specimen surface (including images of the fractured sample) in bone cements containing 5 wt % of nanoparticles at different core-shell ratios. For comparative purposes, the data that were obtained from the bone cement prepared without nanoparticles were also presented. In these tests, only the AE signals that were recorded between inner loading points were analyzed (Figure 2). It can be seen that all of the bone cement formulations prepared with core-shell nanoparticles emitted a higher number of hits during the four-point bending test in comparison to those signals that were presented in the reference bone cement. These results suggested that the detected hits were mainly attributed to the presence of nanoparticles in the bone cement, because the reference bone cement only emitted AE signals of high amplitude and energy around the final fracture (Figure 4a). 

Apparently, the core-shell ratio played an important role on the fracture mechanism of the bone cement, since a slight increase in the total AE signals activity was observed in the bone cements that were prepared with core-shell nanoparticles 20/80 and 30/70 (Figure 4b,c) in comparison to those signals that were obtained in the bone cements containing core-shell nanoparticles 40/60 and 50/50 (Figure 4d,e). This was attributed to the fact that the core-shell nanoparticles 20/80 and 30/70 promoted a higher number of microcracks initiation and propagation sites into the material when compared to the other formulations. 

**Figure 5:**
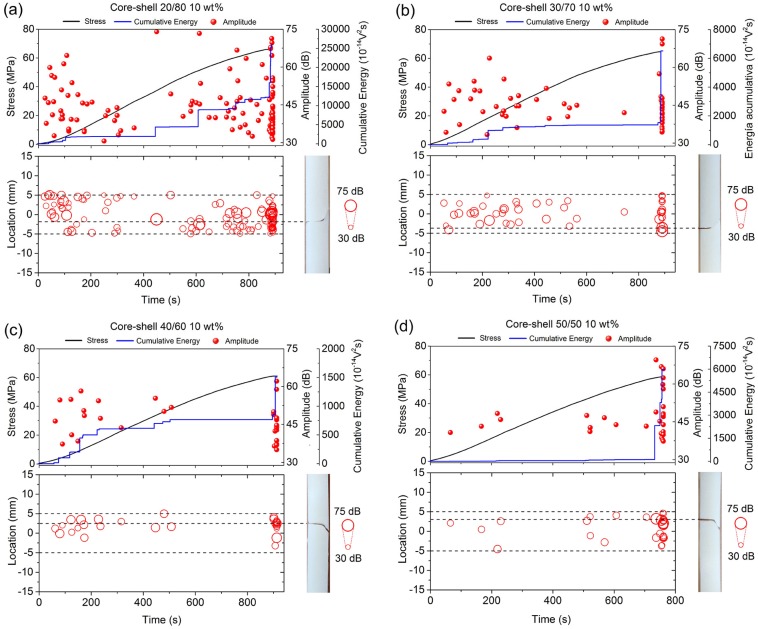
AE signals detected during the four-point bending test in correlation to their respective locations along the specimen surface in the bone cements containing 10 wt % of core-shell nanoparticles (**a**) 20/80, (**b**) 30/70, (**c**) 40/60, and (**d**) 50/50.

Figure 5 shows that the AE signals detected in the bone cements prepared with 10 wt % of core-shell nanoparticles exhibited a similar tendency to those AE signals that were observed in the previous tests, which indicated an increase in the total acoustic activity of the formulations that were prepared with core-shell nanoparticles 20/80 and 30/70 (Figure 5a,b). Additionally, it was observed that, for all bone cement formulations, a high number of AE signals were mainly registered from beginning of the test, approximately from 25 s. It has been suggested that these signals were due to the existence of initial damages in the material, which were retained until the final fracture started to propagate.

**Figure 6:**
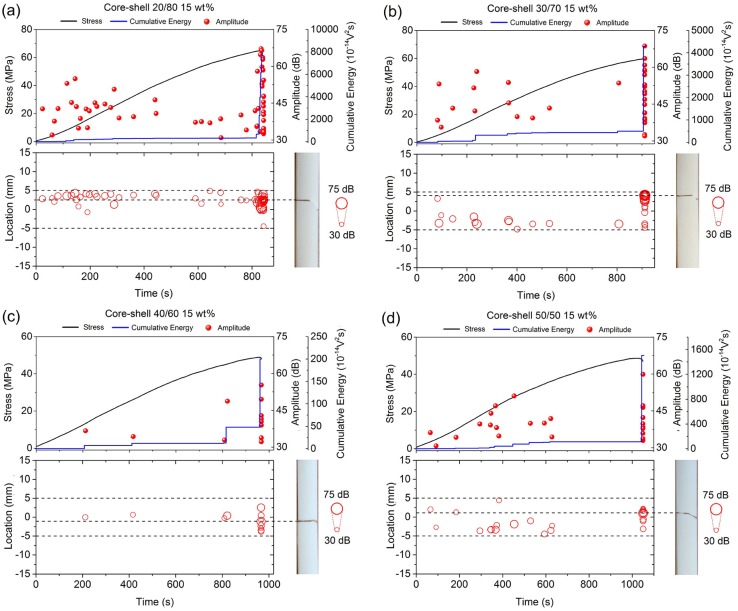
AE signals detected during the four-point bending test in correlation to their respective locations along the specimen surface in the bone cements containing 15 wt % of core-shell nanoparticles (**a**) 20/80, (**b**) 30/70, (**c**) 40/60, and (**d**) 50/50.

Furthermore, Figure 6 displays the data that were obtained from the bone cements containing 15 wt % of core-shell nanoparticles. Consistent with the previous cases, bone cements containing the nanoparticles 20/80 and 30/70 presented the higher acoustic activity of all formulations and the damage also initiated mainly from beginning of the test (time > 25 s). However, the lower number of acoustic signals, presenting in the formulations containing the nanoparticles 40/60 and 50/50 (Figure 6c,d), has been attributed to the fact that they promoted a lower number of nucleation sites for microcracks into the cements.

In the bending test, the cross-section of a specimen is subjected to tension and compression on two sides of its neutral plane (the upper part, concave side, is in compression and the lower part, convex side, is in tension); the plastic deformation on the tensile side starts much earlier than the compression side [23]. Thus, the AE signals source that were recorded in all bone cement formulations mainly came from the tensile side of the specimen, covering the acoustic signals emitted by the compression side.

The increase in the total acoustic activity for the formulations that were prepared with core-shell nanoparticles could be explained if it is considered that the addition of these nanoparticles promotes different mechanisms of energy absorption, as reported by Vila et al. [24] in acrylic bone cement that was modified with the elastomeric copolymer acrylonitrile-butadiene-styrene (ABS). This is, the nanoparticle agglomerates have a double effect on the bone cement; firstly, the elastomeric particles promote additional nucleation sites for microcracks or crazes and, secondly, they can also hinder the propagation of these defects, acting as a barrier to their growth. It is thought that during the four-point bending mechanical test, crazes, or microcracks tend to be initiated in the sites with maximum stress concentration, which are generally near of the vicinity of the nanoparticle agglomerates. Therefore, a higher number of crazes or microcracks are presented in the bone cements containing nanoparticles, which are reflected as an increase in their total acoustic activity.

On the other hand, the higher number of hits detected in the formulations that were prepared with core-shell nanoparticles 20/80 and 30/70 has been explained to the higher capacity of these elements to hinder the crazes or microcracks propagation by presenting a better interfacial adhesion with the acrylic matrix of bone cement; because they have a larger shell thickness than the nanoparticles 40/60 and 50/50. Consequently, in the bone cement that was modified with nanoparticles 20/80 and 30/70 more and shorter crazes or microcracks can be formed when compared to the other formulations, which need more energy to break up the nanoparticle agglomerates to continue growing along the matrix. Therefore, this fact leads to an increase of the absorbed energy and explains the rise of hits emitted during the tests.

### 3.3. Microscopy Analysis

**Figure 7:**
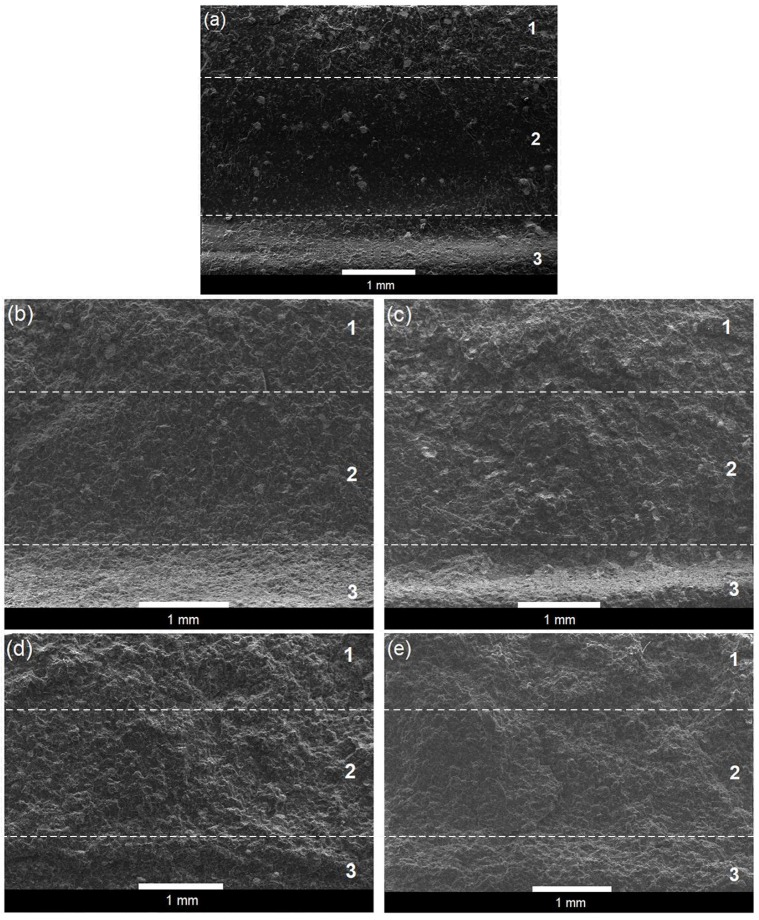
Scanning Electron Microscopy (SEM) micrographs of the bending fracture surfaces of the (**a**) reference bone cement and the bone cements prepared with 15 wt % of structured nanoparticles (**b**) 20/80, (**c**) 30/70, (**d**) 40/60, and (**e**) 50/50. Number 1 indicate the compression band, number 2 the transition band, and number 3 the tensile band.

Figure 7 showed the SEM images of the fracture surfaces that were obtained at the end of the four-point bending tests for the bone cements prepared with 15 wt % of structured nanoparticles at different core-shell ratios. For comparative purposes, the fracture surface that was obtained from the bone cement prepared without nanoparticles is also presented. The direct examination of the fracture surfaces revealed that all bone cement formulations exhibited stress whitening, which arises from the development of crazes before the crack propagation. It is also shown that the fracture surface of the reference bone cement presented three bands with different textural features: a compression band (band 1), a transition band (band 2), and a tensile band (band 3). This was similar to that reported by Liu et al. [23], who also observed three bands with different features on the bending fracture surfaces in four commercial acrylic-based bone cements (Palacos R-40, CMW1 Radiopaque, CMW2000 and Simplex P). The tensile band in the reference bone cement looks rather rough, showing a certain ductile tearing along this surface, where the stress whitening is very notable, as can be seen in Figure 7a. In contrast, in the compression band is appreciated a lower ductility, due to the smoother surface showed in this region. The transition band exhibits a different texture, presenting a less rough surface than the above-mentioned bands.

The SEM images also show that the fracture surfaces of the bone cements containing core-shell nanoparticles (Figure 7b–e) exhibited a higher ductile tearing (higher roughness) in the tensile and compression band; inclusive, they presented a certain degree of roughness in the transition band, which could not be distinguished from the compression band. The degree of roughness presented in the specimens that were prepared with nanoparticles increased as the rubbery core was higher in the core-shell ratio to the point that these bands were not distinguished on the fracture surface of the formulations containing structured nanoparticles 40/60 and 50/50 (Figure 7d,e) for the high roughness presented. The observation of these micrographs suggests a more ductile behavior of the modified bone cement than the reference bone cement.

**Figure 8:**
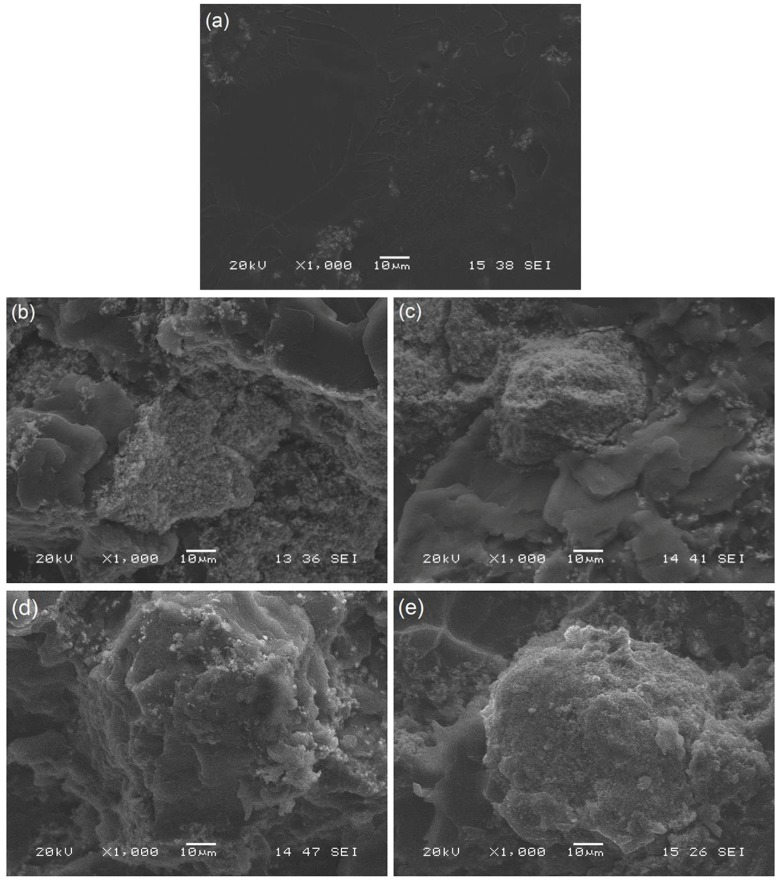
SEM micrographs of the bending fracture surfaces on the transition band at x1000 magnification of the (**a**) reference bone cement and the bone cements prepared with 15 wt % of structured nanoparticles (**b**) 20/80, (**c**) 30/70, (**d**) 40/60, and (**e**) 50/50.

Figure 8 shows a close view of the bending fracture surfaces of the transition band (at x1000 magnification) for the formulations that are presented in Figure 7. It can be seen again that the transition surface on the reference bone cement exhibited a smooth surface, showing small defects which were associated to the presence of BaSO_4_. In contrast, the bone cements that were prepared with core-shell nanoparticles showed a rough surface with certain elements (agglomerates) that were not observed on the fracture surface of the reference bone cement. These defects were attributed to the presence of nanoparticle agglomerates, as they had an average particle size that was very similar to that reported by the authors [14]. 

Based on the above results, it is possible to postulate that introducing the core-shell nanoparticles into the bone cement formulation causes a more ductile behavior when compared to the reference bone cement. On the other hand, the bone cements that were prepared with nanoparticles at 10 wt % concentration offered the higher potential as an alternative to current conventional bone cements due to its tendency to retain more mechanical properties of bone cement.

The fracture mechanism that was proposed for the four-point bending test is very similar to that reported for the quasistatic compression tests [14], as well as for the tensile fatigue tests [15], where the AE signals source mainly arise from the tensile side of the bending specimen. 

## 4. Conclusions

This investigation confirmed the potential of the core-shell nanoparticles to be employed as an alternative method for reducing the bending modulus and bending strength in the bone cement as well as the wide-range potential of the AE technique to localize the fracture on the specimens, with this technology being applicable to other commercial bone cements. It is concluded that, during the bending test in the tensile band of the specimen, crazes or microcracks tend to be initiated near the vicinity of the nanoparticle agglomerates; meanwhile, these elements also tend to hinder the propagation of the defects, preventing their growth. 

The bone cements containing nanoparticles 20/80 and 30/70 emitted the higher number of hits during the bending tests, due to these elements presenting more and shorter crazes or microcracks than the other formulations. Finally, the use of the AE technique and the fracture surface analysis by SEM enabled insight of the damage evolution presented during four-point bending test of the bone cements containing nanoparticles.

## Figures and Tables

**Table 1:** Bone cement formulations.

Powder Component				Liquid Component	
Core-Shell Nanoparticles	PMMA	BaSO_4_	BPO	MMA	DMPT
0 ^1^	89	10	1	97.5	2.5
5	84	10	1	97.5	2.5
10	79	10	1	97.5	2.5
15	74	10	1	97.5	2.5

^1^ Reference bone cement. Compositions are in percent (*w*/*w*).
